# Single-Base Pair Genome Editing in Human Cells by Using Site-Specific Endonucleases

**DOI:** 10.3390/ijms160921128

**Published:** 2015-09-03

**Authors:** Hiroshi Ochiai

**Affiliations:** Research Center for the Mathematics on Chromatin Live Dynamics (RcMcD), Hiroshima University, Higashi-Hiroshima 739-8530, Japan; E-Mail: ochiai@hiroshima-u.ac.jp; Tel.: +81-82-424-5568 (ext. 5568); Fax: +81-82-424-5529.

**Keywords:** single-nucleotide polymorphisms, single-base pair editing, genome editing, programmable nucleases, zinc-finger nuclease, TALEN, CRISPR, gene therapy

## Abstract

Genome-wide association studies have identified numerous single-nucleotide polymorphisms (SNPs) associated with human diseases or phenotypes. However, causal relationships between most SNPs and the associated disease have not been established, owing to technical challenges such as unavailability of suitable cell lines. Recently, efficient editing of a single base pair in the genome was achieved using programmable site-specific nucleases. This technique enables experimental confirmation of the causality between SNPs and disease, and is potentially valuable in clinical applications. In this review, I introduce the molecular basis and describe examples of single-base pair editing in human cells. I also discuss the challenges associated with the technique, as well as possible solutions.

## 1. Introduction

Programmable nucleases, including zinc-finger nucleases (ZFNs), transcription activator like-effector nucleases (TALENs), and Clustered Regulatory Interspaced Short Palindromic Repeats (CRISPR)/CRISPR-associated 9 (Cas9), may be used to engineer double-strand breaks (DSBs) in the genome. By then exploiting the endogenous DSB repair pathway in cells, genomes can be edited to disrupt, introduce, invert, delete, or correct genes [1]. Genome editing with ZFNs and TALENs initially attracted much attention, but was not widely adopted because of the complexity in designing these enzymes. The introduction of CRISPR/Cas9 in 2013 [2,3] has dramatically changed life science research in many ways [4], because the system is significantly easier to implement. Genome editing is now used in medical research to, for example, identify causal mutations underlying inherited disorders [5], establish disease models via induced pluripotent stem (iPS) cells [6], and treat human immunodeficiency virus infection or acquired immune deficiency syndrome [7].

Genome-wide association studies have identified a large number of single-nucleotide polymorphisms (SNPs) associated with human disorders and with physical traits such as height, olfactory sensitivity, and skin color [8]. SNPs in coding regions or splice sites that are predicted to drastically change protein structure or function are suspected, with good reason, to underlie the associated disorder or trait. In these instances, functional analysis of the SNP is relatively straightforward. However, SNPs that are intergenic or non-coding are not as easily characterized [9]. On the other hand, high-throughput DNA sequencing has enabled identification of actively regulated regions that are enriched in specific histone modifications, transcription factor binding sites, or DNase-hypersensitive sites [10]. SNPs within these putative regulatory sequences, aptly named regulatory SNPs, are expected to reliably produce the observed phenotypes [9,11,12]. Nevertheless, experimental characterization of these SNPs remains challenging [13]. This situation is especially true of single-nucleotide variations strongly associated with rare disorders.

One of the most straightforward methods to characterize SNPs, single-nucleotide variations, or major allelic variants is to engineer them directly into the genome. Unfortunately, single-base pair editing through spontaneous homologous recombination is impractical or unachievable. Therefore, efficient, site-specific genome-editing technologies based on programmable nucleases are required [14]. Seamless genome editing, in which target nucleotides are mutated without further footprint, is also critical, especially in regenerative medicine [15]. Importantly, the efficiency of seamless genome editing largely depends on the system and technique used.

In this review, I will focus on single-base pair editing and related techniques, especially those used for seamless genome editing in human cells. I will also examine the merits and demerits of each technique, and explore potential technical improvements.

## 2. Seamless Single-Base Pair Editing and Related Techniques

**Figure 1:**
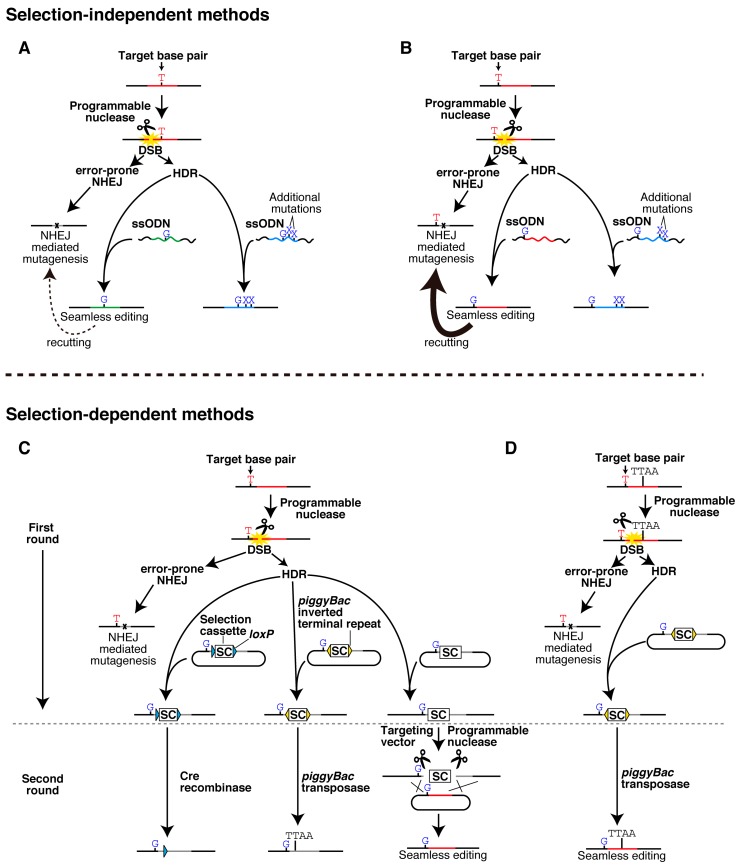
Single-base pair genome editing and related techniques. Schematic representation of methods to engineer a single or a small number of nucleotide substitutions into the genome. (**A**,**B**) Selection-independent editing of a target base pair within (**A**) and out (**B**) of a sequence recognized by a programmable nuclease; (**C**,**D**) Selection-dependent editing using different methods to excise the selection marker. *piggyBac*-excision is illustrated without (**C**) or with (**D**) a TTAA sequence naturally present near or at the target site. Blue, gray, red, and green lines represent programmable nuclease target sites with deletions, insertions, single, and multiple nucleotide substitutions, respectively. Target sites with single nucleotide substitutions may be recut by the programmable nuclease depending on the properties of the enzyme and the location of the substitution. Scissors and yellow star shapes represent programmable endonucleases and DNA double-strand breaks (DSBs), respectively.

Seamless single-base pair editing and related techniques require highly site-specific programmable nucleases, and exploit homology-directed repair (HDR), one of two major endogenous DSB repair pathways (Figure 1). These techniques are categorized into two classes based on whether selection markers are used.

In selection-independent genome editing, only the intended nucleotide substitutions are directly engineered into the target site via a DSB and a template that consists of either plasmids or single-stranded oligodeoxynucleotides (ssODNs) (Figure 1A,B, and Table 1). However, HDR is less frequently activated in cells than non-homologous end joining (NHEJ), the other major DSB repair pathway, which is error-prone and frequently introduces additional deletions and insertions. Therefore, appropriate methods are required to identify cells with only the desired nucleotide changes (Figure 1).

On the other hand, selection-dependent methods require two rounds of genome editing (Figure 1C,D, and Table 2). In the first round, a plasmid vector is used to knock nucleotide substitutions into the target site, along with selection markers. These selection markers are then excised in the subsequent round (Figure 1C,D). While this method seems more labor-intensive, it is also more efficient and reliable.

**Table 1:** Selection-independent seamless genome editing.

Programmable Nuclease	Host Cell	Target	Template	Edits Introduced	Reference
ZFN	K562	*IL2Rγ*	plasmid	1 bp substitution	[14]
	CD4+ T				
ZFN	K562	*RSK2*	ssODN	Substitution of 6 bp in and out of ZFN recognition site	[16]
ZFN	ES	*SNCA*	ssODN	1 bp substitution	[17]
	iPS		plasmid		
TALEN	iPS	*CCR5*	ssODN	2 bp substitutions	[18]
CRISPR					
TALEN	iPS	*AKT2*	ssODN	2 bp substitutions	[6]
TALEN	iPS	*PHOX2B*	ssODN	1 bp substitution	[19]
		*PRKAG2*			

**Table 2:** Selection-dependent seamless genome editing.

Programmable Nuclease	Excision Method	Host Cell	Target	Edits Introduced	Reference
ZFN	Cre/*loxP*	ES iPS	*SNCA*	1 bp substitution and *loxP* site insertion	[17]
ZFN	*piggyBac*	iPS	*A1AT*	1 bp substitution of interest and 2 to generate TTAA site	[20]
CRISPR	*piggyBac*	iPS	*HBB*	1 bp substitution and 4 bp insertion *	[15]
TALEN	*piggyBac*	iPS	*HBB*	3 bp substitutions	[21]
TALEN	TALEN	HCT116	Interge region (upstream of *BUBR1*)	1 bp substitution	[5]

* Two different *β-thalassemia* mutations (a single nucleotide substitution and a 4 bp deletion) were corrected in two different alleles. Therefore, the correction is a single-base pair edit and a 4-bp insertion in each allele.

### 2.1. Selection-Independent Methods

Selection-independent methods are straightforward, because nucleotide substitutions are engineered in a single step without the use of selection markers (Figure 1A). In addition, ssODNs may be used as template instead of plasmids [16,17], which could be randomly integrated into the host genome [16]. Nevertheless, the use of highly specific programmable nucleases is critical to minimize recutting and insertion of unintended mutations by NHEJ (Figure 1A,B). ZFNs with high specificity may distinguish a single nucleotide substitution [22]. However, these enzymes are limited in the range of sequences they can target, and, therefore, an appropriate ZFN may not be available for a target base pair [17]. In this case, additional mutations will have to be introduced to inhibit recutting and mutagenesis by NHEJ [16] (Figure 1A). For target sites in coding regions, synonymous mutations that inhibit recutting may not be an issue. However, many SNPs are, as noted, in non-coding regions of unknown function, to which introduction of a neutral substitution is difficult, if not impossible. Unfortunately, preparation of ZFNs with the desired specificity is extremely labor-intensive. As a result, these nucleases are not widely used in academic research [23].

On the other hand, TALENs are much easier to obtain, and are almost unrestricted in targetable sites [24]. These sites are relatively long (~17 bp each), and very high specificity can be achieved at unique or sufficiently distinctive targets. However, TALENs, in most cases, cannot distinguish a single nucleotide mismatch. Thus, TALENs are not suitable for seamless genome editing by selection-independent methods [18]. On the other hand, the widely used *S. pyogenes* Cas9 (SpCas9) has been demonstrated to detect a mismatched nucleotide near or in the protospacer adjacent motif (PAM) [2,25]. Therefore, if the target base pair is located near or in this motif, CRISPR/Cas9 can be useful to seamlessly edit single base pairs without selection.

The identification and isolation of cells with the intended nucleotide changes are a major challenge. In general, exogenous DNA and RNA are not delivered uniformly into individual cells, and some cells are not transduced at all. Conversely, cells receiving a high dose may become unviable [26,27]. To avoid this problem, site-specific endonucleases are delivered at a small dose, along with template DNA and a vector expressing either a drug-resistance protein or green fluorescent protein. Transformed cells are then identified by transient drug selection or fluorescence-activated cell sorting, respectively [6,17,28]. This approach drastically enhances efficiency.

Recently, Miyaoka *et al.* [19] described an alternative screening method. In this approach, TALEN expression vectors are delivered at low dose to minimize off-target effects. Then, cells are replated into a 96-well plate. After the incubation, cells are split into two replicate cultures, one of which is analyzed by highly sensitive digital PCR for the desired nucleotide substitution. Cells from the remaining replicate well that contains the desired edits are further replated, multiple times if necessary, to purify transformed cells. In the end, a cell line with a single nucleotide substitution is established.

### 2.2. Selection-Dependent Methods

Although selection-dependent methods are labor-intensive, and require two rounds of editing (Figure 1C,D and Table 2), they may be ultimately more efficient. The intended nucleotide substitution is not always integrated into the genome, and insertion efficiency depends to a significant extent on the distance between the desired mutation and the cut site of the programmable nuclease [29] (Ochiai, data not shown). The use of markers, including puromycin resistance for positive selection and herpes simplex virus thymidine kinase for negative selection, enables efficient selection of cell clones that contain the desired mutations [20]. Thus, nucleotide substitutions of interest are engineered into the target site along with a selection cassette in the first round. To avoid recutting, the selection cassette is typically inserted within the endonuclease target site. In the second round, the selection cassette is excised, usually by the Cre/*loxP* system [17], by the *piggyBac* system [15,20,29], or by a programmable nuclease [5].

#### 2.2.1. Cre/*loxP*-Mediated Excision

The Cre-*loxP* system is a long-established tool for genetic manipulation, and enables deletions, insertions, translocations, and inversions at specific genomic sites in cells [30]. Therefore, the selection cassette can be easily excised by expression of Cre recombinase if it is flanked by 34-bp *loxP* sites [17]. However, a *loxP* site is left behind as a footprint and may confound the effects of the intended substitutions (Figure 1C). Furthermore, the system also has off-target concerns as programmable nucleases [31,32].

#### 2.2.2. *piggyBac*-Mediated Excision

*piggyBac* transposons are flanked by *piggyBac* inverted terminal repeats, and transpose between vectors and chromosomes in a process that requires *piggyBac* transposase [33,34,35]. Excision of the transposon leaves a TTAA fragment behind, a scar that may, as noted, complicate the interpretation of results (Figure 1C). However, if a TTAA site is naturally present at or near the target site, *piggyBac*-mediated genome editing is essentially seamless (Figure 1D) [15].

#### 2.2.3. Excision by a Programmable Nuclease

Programmable nucleases may also be used to excise the selection cassette out of the target site. In this method, a secondary targeting vector is required, as well as additional programmable nucleases that will cut sequences that flank the selection cassette (Figure 1C) [5]. Alternatively, secondary ssODNs may also be used instead of secondary plasmids [16]. On the other hand, the need for a secondary vector may be eliminated entirely by adding microhomology sites at both sides of the selection cassette to enable efficient excision via microhomology-mediated DSB repair [36].

Programmable nucleases circumvent the need for a TTAA fragment near the target site, as would be required in *piggyBac*-mediated excision. However, the need to design these additional programmable nucleases is a drawback, as is the additional risk of off-target effects from such secondary enzymes. The use of CRISPR enzymes instead of ZFNs and TALENs may help reduce the labor required, while off-target effects may be minimized with ZFNs and TALENs that contain heterodimeric nuclease domains [37,38,39] or with Cas9 nickase mutants [40].

## 3. Conclusions

Genome editing with programmable nucleases enables efficient, seamless substitutions of a single or a small number of nucleotides at predefined sites. However, technical hurdles remain, and prevent widespread adoption. To date, the CRISPR/Cas9 system is the most attractive, for ease of implementation and ability to distinguish a single mismatched nucleotide in or near the PAM sequence. Recently, several Cas9 orthologs from different species have been reported, as well as SpCas9 mutants that recognize an array of PAM sequences [41,42]. Furthermore, the structure of Cas9 has been determined, and I anticipate that Cas9 mutants with diverse PAM sequence specificities will soon be identified or engineered [43,44]. Indeed, single-nucleotide editing with ssODN templates will become highly efficient if programmable nucleases could be engineered to have broad targetable sequences, high specificity, and sensitivity to single-nucleotide mismatches.
